# Incidence of Dravet Syndrome in a US Population

**DOI:** 10.15844/pedneurbriefs-29-12-3

**Published:** 2015-12

**Authors:** Jena Krueger, Anne T. Berg

**Affiliations:** 1 Division of Epilepsy, Ann & Robert H. Lurie Children's Hospital of Chicago, Chicago, IL

**Keywords:** Epilepsy, Genetics, Pediatrics

## Abstract

Investigators from the University of California, San Francisco and Kaiser Permanente report the incidence of Dravet Syndrome in a population based cohort.

Investigators from the University of California, San Francisco and Kaiser Permanente report the incidence of Dravet Syndrome in a population based cohort. The cohort was identified by reviewing records of patients treated in Kaiser Permanente Northern California (KPNC) system from January 1, 2007 to June 30, 2010. KPNC has a catchment area with over 3.5 million members and consists of approximately half of the insured population in Northern California.

The study was a retrospective chart review of all infants born within the KPNC system during the specified timeframe. Records were reviewed and a clinical diagnosis of Dravet Syndrome was made if the patient met 4 of the 5 following criteria: 1. Normal or near normal cognition prior to onset, 2. Two or more febrile or afebrile seizures in the first year of life, 3. Myoclonic, hemiclonic or generalized tonic-clonic seizures, 4. Two or more seizures lasting longer than 10 minutes, 5. Failure to respond to a first-line antiepileptic medication with continued seizures after two years of age. All patients who met the clinical diagnosis criteria for Dravet Syndrome had SCN1A gene sequencing performed.

There were 125,547 births in the study population, of which 730 infants had two or more seizure visits prior to the age of one year. Eight infants met 4 of the 5 clinical criteria for Dravet Syndrome, equivalent to an incidence of 1 per 15,700 births). Six of the eight patients were found to have de novo mutations that were predicted to be pathogenic, equivalent to an incidence of 1 per 20,900. [1]

COMMENTARY. Dravet Syndrome is a rare pediatric epilepsy syndrome encompassing a range of cognitive delays and refractory epilepsy. The authors provide estimates of its incidence based on well-defined clinical features ascertained in the first year of life and on genetic testing. Although the incidence of Dravet Syndrome was somewhat higher than others have found, the percentage of Dravet patients with a SCN1A mutation (75%) is similar to previous reports. Early diagnosis of Dravet Syndrome is important, as it may guide providers in selecting drugs demonstrated to be most effective and in steering away from drugs known to exacerbate seizures in Dravet Syndrome. Patients with suspected Dravet Syndrome and other pediatric refractory epilepsy could benefit greatly from earlier diagnosis, potentially provided by early genetic testing.

Ream and Mikati at Duke University medical center reported on their pilot program for genetic testing in new epilepsy patients. Twenty-five pediatric patients with refractory epilepsy completed genetic testing, of which 15 had a positive result. Patients with generalized epilepsy or epileptic encephalopathy were more likely to have a pathogenic variant. Of the different methods, karyotype testing had the lowest yield (1 out of 7 patients tested, 14.3%), while epilepsy gene panel testing had the highest yield (6 of 13 patients tested, 46.2%). Of the six patients tested with whole exome Sequencing, 100% had pathogenic variants, although only one of the six patients had a specific diagnosis as a result of the testing [2]. Mercimek-Mahmutoglu et al. at The Hospital for Sick Children found 28% of patients in a cohort of patients referred for epileptic encephalopathy had an identifiable genetic cause [3].

As genetic testing becomes more refined and available the opportunity for earlier diagnosis and treatment will become greater. Multicenter collaboration will be essential for developing optimal approaches for the diagnosis, treatment and management of Dravet Syndrome and other rare epilepsies.
